# Supplementary material for: ONC201 Suppresses Neuroblastoma Growth by Interrupting Mitochondrial Function and Reactivating Nuclear ATRX Expression While Decreasing MYCN
Source: Int J Mol Sci. 2023 Jan 13;24(2):1649. doi: 10.3390/ijms24021649 (PMC9867473; doi:10.3390/ijms24021649)
Supplement: Supplementary file 1 [file ijms-24-01649-s001.zip › ijms-1903210-supplementary.pdf]

Supplementary information

ONC201 Suppresses Neuroblastoma Growth by Interrupting Mitochondrial Function and Reactivating of Nuclear ATRX Expression While Decreasing MYCN

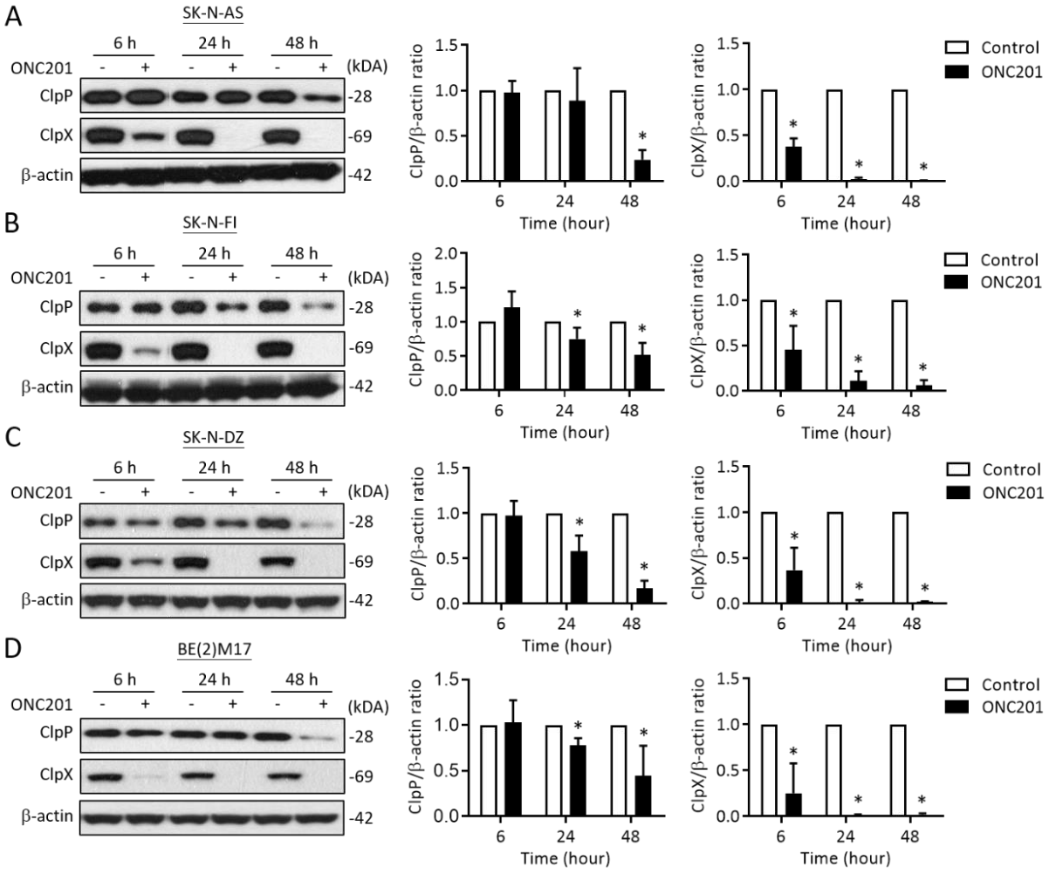

Figure S1. Effect of ONC201 on the ClpP and ClpX expression in time-dependent manner in NB cells. (A-D) After incubation with ONC201 (5 μM) at indicated time-point, cells were harvested for following protein analysis. The relative protein levels of ClpP and ClpX were determined by Western blot and quantified by Image Pro-plus analysis software. Data were calculated as fold change ± SD from triplicate experiments. *: $p < 0.05$.

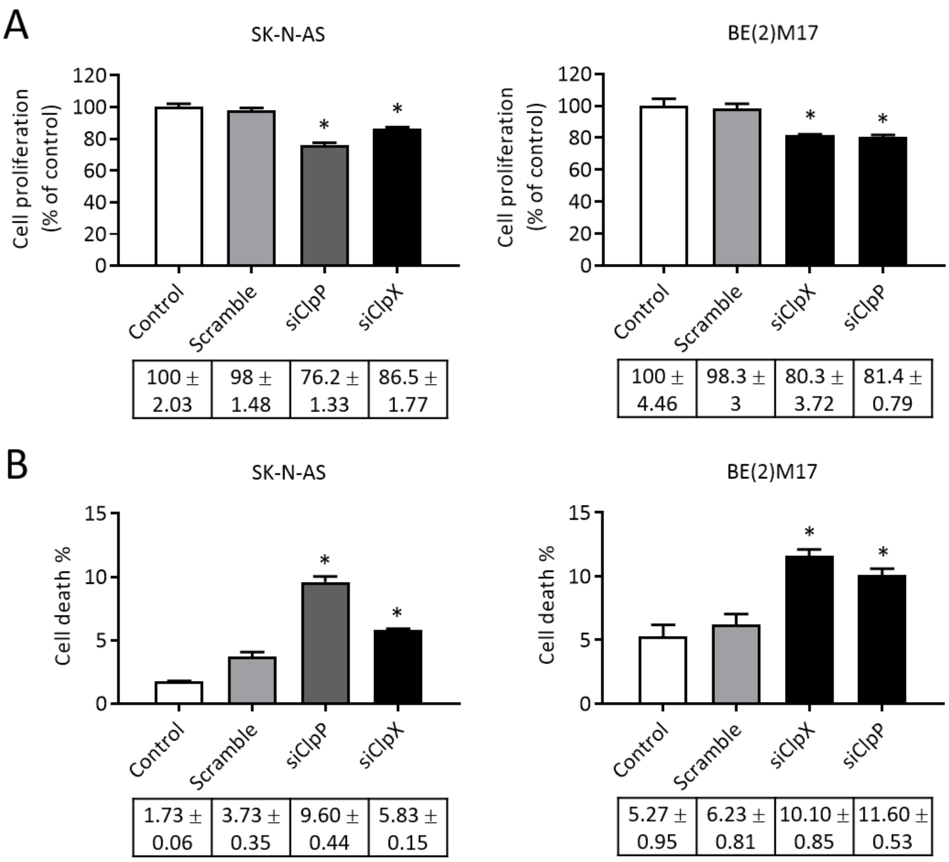

Figure S2. Effects of ClpPX complex interruption on cell survival in NB cells. Cell proliferation assay (A) and cell death (B) after siClpP and siClpX treatment of SK-N-AS and BE(2)M17 NB cells. All data were calculated and expressed as mean ± SD from triplicate experiments. *: $p < 0.05$.

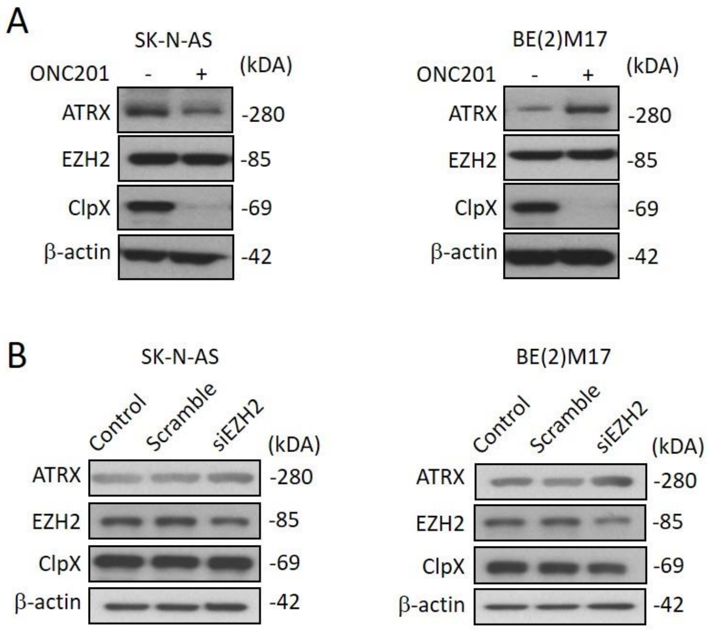

Figure S3. Western blot analysis of ATRX and EZH2 expression in ONC201 treatment and EZH2 siRNA interference of NB cells. (A) NB cells were treated with ONC201 (5 μM) for 48 h before Western blot analysis. ONC201 treatment did not affect the expression of EZH2 in SK-N-AS and BE(2)M17 cells, despite ATRX was increased in BE(2)M17, but decreased in SK-N-AS . (B) NB cells were transfected with scramble or siEZH2 using Lipofectamine 3000 Reagent and incubated with complete medium for 48 h. EZH2 gene silence mildly promoted ATRX expression in BE(2)M17 cells, not in SK-N-AS cells.

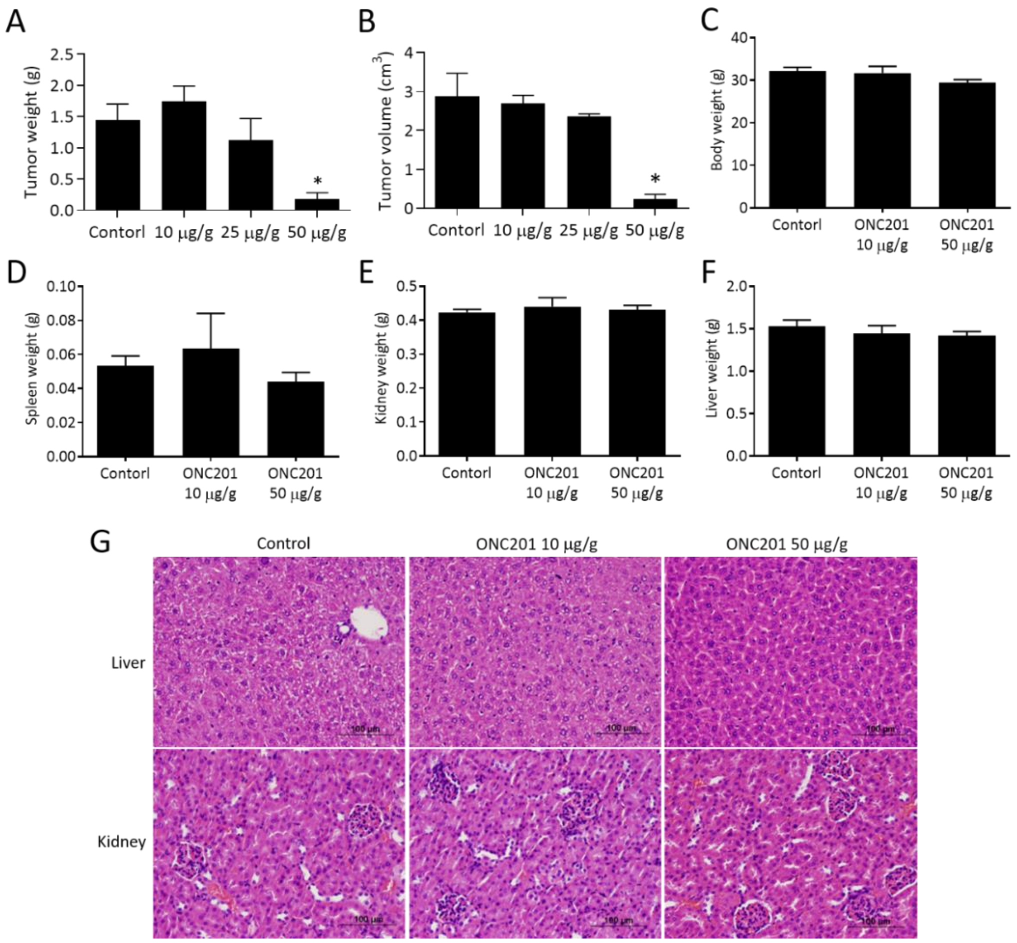

Figure S4. Effect of ONC201 treatment on tumor growth, body and organ weight of mice and histopathology of liver and kidney. (A and B) Tumor weight (g) and tumor volume (cm^3) were measured at sacrifice by microbalance and expressed as mean ± SD. (C-F) Mice body and organ weights were measured by microbalance at the end of the experiment. There was no significant difference between groups, despite a trend toward body weight loss in mice receiving ONC201. Bar chart was expresses as mean ± SD. Control = 3, treatment = 5. (G) Representative HE staining of the liver and kidney tissues of control and ONC201-treated mice, which showed no significant changes between groups. Scale bar, 100 μm.
